# Enhancing Ligand and Protein Sampling Using Sequential Monte Carlo

**DOI:** 10.1021/acs.jctc.1c01198

**Published:** 2022-05-19

**Authors:** Miroslav Suruzhon, Michael S. Bodnarchuk, Antonella Ciancetta, Ian D. Wall, Jonathan W. Essex

**Affiliations:** † School of Chemistry, University of Southampton, Highfield, Southampton SO17 1BJ, U.K.; ‡ Computational Chemistry, R&D Oncology, AstraZeneca, Cambridge CB4 0WG, U.K.; § Sygnature Discovery, Bio City, Pennyfoot Street, Nottingham NG1 1GR, U.K.; ∥ Department of Chemical, Pharmaceutical and Agricultural Sciences—DOCPAS, University of Ferrara, Via Fossato di Mortara 17/19, 44121 Ferrara, Italy; ⊥ GSK Medicines Research Centre, Gunnels Wood Road, Stevenage SG1 2NY, U.K.

## Abstract

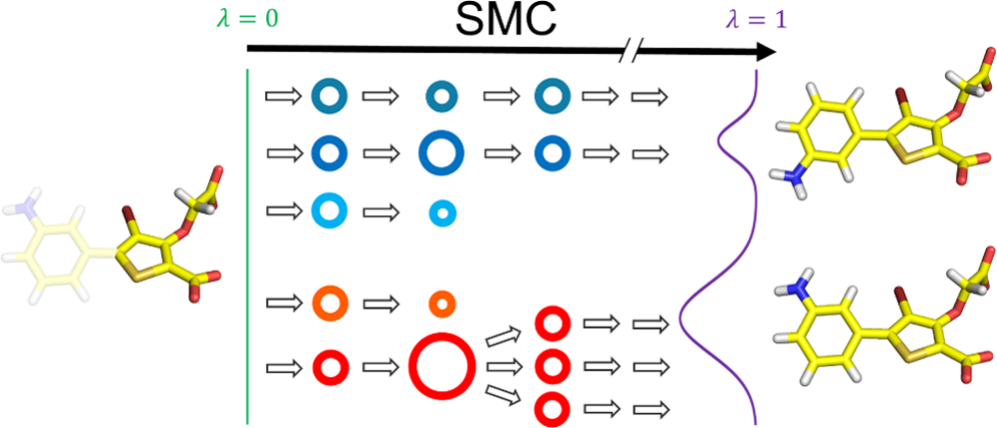

The sampling problem is one of the most widely studied topics in computational chemistry. While various methods exist for sampling along a set of reaction coordinates, many require system-dependent hyperparameters to achieve maximum efficiency. In this work, we present an alchemical variation of adaptive sequential Monte Carlo (SMC), an irreversible importance resampling method that is part of a well-studied class of methods that have been used in various applications but have been underexplored in computational biophysics. Afterward, we apply alchemical SMC on a variety of test cases, including torsional rotations of solvated ligands (butene and a terphenyl derivative), translational and rotational movements of protein-bound ligands, and protein side chain rotation coupled to the ligand degrees of freedom (T4-lysozyme, protein tyrosine phosphatase 1B, and transforming growth factor β). We find that alchemical SMC is an efficient way to explore targeted degrees of freedom and can be applied to a variety of systems using the same hyperparameters to achieve a similar performance. Alchemical SMC is a promising tool for preparatory exploration of systems where long-timescale sampling of the entire system can be traded off against short-timescale sampling of a particular set of degrees of freedom over a population of conformers.

## 1 Introduction

The sampling problem presents one of the biggest challenges in the field of classical computational chemistry, particularly biomolecular simulation.^1,2^ Since current computational power is insufficient for studying statistical mechanical problems of systems with more than 10,000 atoms at the relevant millisecond to minute timescales, enhanced sampling methods have been an indispensable tool in the computational chemist’s arsenal, trading dynamic detail for the sampling of relevant rare transitions at short timescales.

More generally, the enhanced sampling problem can be referred to as the multimodal global sampling problem, that is, sampling from a probability distribution with multiple relevant modes (i.e., highly populated states), which are highly disconnected and whose locations are generally not known a priori. In a typical physical application, multimodality manifests itself through high kinetic barriers, where the probability of surmounting them decreases exponentially with their heights. As a result, many such transitions are practically impossible at the currently achievable computational timescales. Enhanced sampling research encompasses two key components: the optimal choice of important degrees of freedom (collective variables, CVs), which is system-dependent, and the method used to sample these degrees of freedom. There are many such enhanced sampling methods,^1^ some of the most widely known being replica exchange molecular dynamics (REMD),^3−6^ metadynamics,^7−9^ and umbrella sampling.^10^

A common challenge for enhanced sampling is the need for some prior knowledge of the system under study, which manifests itself beyond the need for a relevant CV. For example, REMD and umbrella sampling benefit greatly from an optimal spacing of the intermediate states, and methods involving nonequilibrium switching^11,12^ are most efficient when low-variance pathways are used. An obvious requirement for a robust enhanced sampling method is therefore its ability to adaptively tune itself to the system studied, irrespective of the system complexity.

While there has been a considerable body of work in developing adaptive versions of some of the above methods,^9,13−15^ here, we shift our focus to another promising alternative—sequential Monte Carlo (SMC).^16,17^ With SMC being one of the oldest enhanced sampling algorithms,^16^ it has been rediscovered and further developed in many fields, such as statistics,^18^ robotics,^19^ meteorology,^20^ solid-state physics,^21,22^ and quantum chemistry,^23^ often under different names (particle filtering,^24^ weighted-ensemble annealing,^25^ population annealing,^21^ Rosenbluth sampling,^16^ configurational bias Monte Carlo,^26^ and diffusion quantum Monte Carlo^23^). While SMC has already been used in classical computational chemistry as a way to improve sampling methods utilizing nonequilibrium switching,^27−29^ its usage in biomolecular systems has been mostly restricted to polymer growing and protein folding,^30−32^ and its use with more sophisticated force field models has been underexplored.

Most relevant to this work are the recent publications by Christiansen et al.,^33,34^ where the authors used an adaptive tempered version of SMC to explore peptide conformations using molecular force field models. In this work, we will extend this methodology to an alchemical setting, where instead of uniformly increasing the temperature of the whole system, a small subset of the molecular interactions will be completely decoupled instead. This approach is particularly suitable for exploring specific molecular degrees of freedom of interest and has been utilized in other methods, such as Hamiltonian replica exchange molecular dynamics (H-REMD)^35,36^ and nonequilibrium candidate Monte Carlo,^12,37^ and is closely related to alchemical free energy (AFE) methods.^38^ We will also apply alchemical SMC on a variety of protein–ligand complexes to measure its suitability for handling high-dimensional systems of practical interest.

In the following, we will first present one of the most popular SMC algorithms, sequential importance resampling (SIR).^17^ The original version of SIR is not adaptive and conceptually similar to REMD^3,4^ and simulated tempering.^39,40^ Afterward, we will discuss several modifications to the original method, some of which have been extensively explored in the field of statistics, while others have been derived from physical considerations and nonequilibrium statistical mechanics. These will allow us to apply SMC to practically relevant scenarios. Finally, we will conclude with a variety of test cases, where we will show examples of enhancing torsional angle sampling and ligand binding mode exploration in systems with increasing complexity.

## 2 Fundamentals of SIR

The fundamental assumption behind SIR is that one starts from a distribution that is trivial to sample from (e.g., a uniform distribution). In most practical examples, where the distributions have many correlated dimensions, this is not possible and the initial distribution is chosen so that transitions between a subset of the modes are more likely than in the distribution of interest. Afterward, a population of samples is propagated over a number of intermediate distributions that connect the initial distribution to the final distribution of interest.

The main focus of this work are Boltzmann-like distributions of the form
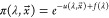
1where *x⃗* are the system coordinates; λ is an adjustable parameter, such that 0 ≤ λ ≤ 1; *u*(λ,*x⃗*) is the dimensionless potential energy of the system, which can also contain additional terms, such as a pressure–volume term in the case of an isothermal–isobaric ensemble; and *f*(λ) is the dimensionless free energy, which normalizes the distribution. The coupling parameter λ is defined to be 0 at the initial distribution and 1 at the final distribution of interest.

**Figure 1:**
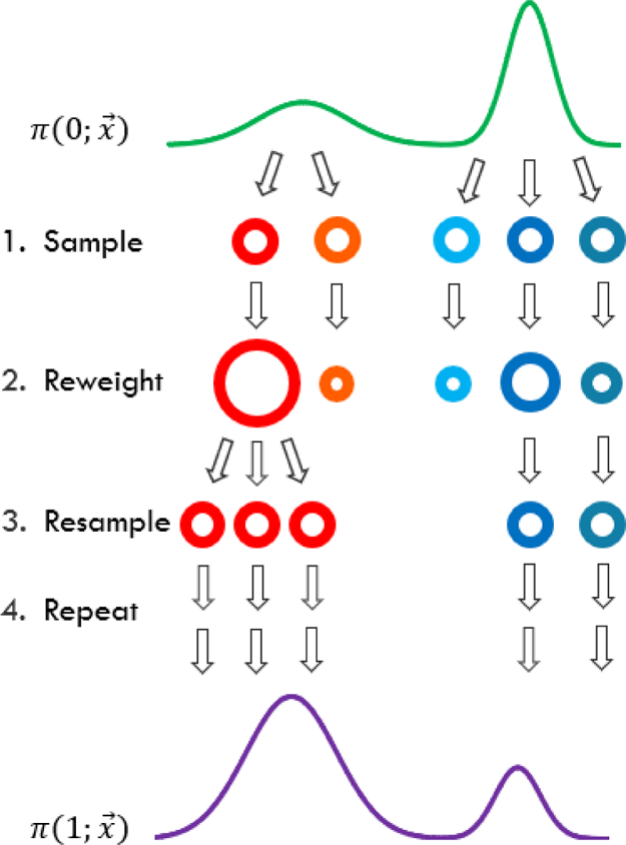
Three stages of each SIR iteration: sampling, reweighting, and resampling. Each unique walker is shown with a different color, and the size of the walker represents its weight. Here, π(0,*x⃗*) and π(1,*x⃗*) represent the initial and final distributions, respectively.

Each SIR iteration consists of three steps (Figure 1): sampling, reweighting, and resampling. Any valid samplers can be used in the first step, such as Markov chain Monte Carlo (MCMC) or Langevin molecular dynamics (MD), to generate a population of *N* locally decorrelated samples (walkers). The second step determines the relative transition probability of the *j*-th walker *p*(λ_*i*+1_|λ_*i*_,*x⃗*_*j*_) between the current distribution π(λ_*i*_,*x⃗*) and the next distribution in the sequence π(λ_*i*+1_,*x⃗*), 0 ≤ λ_*i*_ < λ_*i*+1_ ≤ 1. These relative transition probabilities are normalized and converted into importance sampling weights *w*_*j*_(λ_*i*+1_|λ_*i*_), which are then assigned to each walker
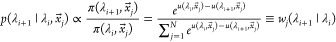
2

The final step of an SIR iteration consists of weighted resampling with replacement based on these weights to generate a new set of equally weighted *N* walkers. This results in the high-weight walkers being copied multiple times and the low-weight walkers being annihilated. This three-step procedure is then repeated for each consecutive distribution until the final distribution has been reached.

One can readily see what sets SIR apart from other enhanced sampling methods: the “survival of the fittest” approach combined with the lack of reversibility and the fact that the method does not satisfy the rather restrictive detailed balance condition, meaning that SIR only explores the best paths and that one can “peek into the future” and adapt the hyperparameters of the method based on this knowledge. This notwithstanding, SIR satisfies a more general stationarity condition, balance,^41^ and is known to be completely rigorous in terms of preserving the target distribution π(1,*x⃗*) in the limit of infinite walkers and infinite sampling at π(0,*x⃗*).^42^ This makes it an asymptotically valid sampling method, similar to all other sampling methods requiring an infinite amount of samples for convergence (e.g., all methods utilizing MD and/or MCMC).

It can be shown that the expectation value of the unnormalized weights  of the samples generated from π(λ_*i*_,*x⃗*) is an unbiased estimator of the partition function ratio  (Zwanzig equation^38^). This means that *Z*(1)/*Z*(0) can also be estimated in an unbiased way from the products of the consecutive expectation values of the unnormalized weights. If one is interested in obtaining unbiased expectation values over separate SIR runs, then the final samples from each run need to be reweighted by the total estimated  for this run,^43^ which can be interpreted as the collective relative weight of the final samples. In effect, the samples are weighted by their free energies, as reflected in the partition function ratio. In this case, the unbiased expectation value ⟨*O*⟩ of an observable *O* over *K* independent SIR simulations, each having *M* walkers, is
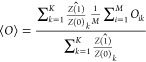
3where *O*_*ik*_ is the observable evaluated on the *i*-th walker in the *k*-th simulation and  is the estimated collective walker weight of the *k*-th simulation.

It is known that this sample reweighting procedure is not in general unbiased for adaptive SIR, where the strides in the λ space depend on the weights at each step.^44^ Although this condition can be circumvented by running adaptive SIR once and using the derived protocol for all consecutive repeats,^45^ this approach is not practical for running simulation repeats in parallel, and in this study, we will apply the reweighting procedure during analysis regardless of this and demonstrate its sufficient precision in a wide range of test cases.

## 3 Adaptive Alchemical SMC

This section highlights some important considerations about performing SMC on a protein–ligand system, as well as several changes to the base method, most of which have been previously considered in the literature.^33,34,46^ Some of these modifications allow us to substitute the system-dependent hyperparameters (e.g., the exact sequence of optimal intermediate distributions) with system-independent hyperparameters (e.g., adaptively choosing the intermediate distributions based on a constant distribution overlap).

### 3.1 Alchemical Perturbation versus Tempering

Enhancing sampling in the temperature space is valuable when one wants to treat all degrees of freedom equally. However, this approach becomes less feasible for large systems, and enhancing specific degrees of freedom is often more desirable whenever possible. In this work, we consider systems where some degrees of freedom are of greater interest than others. For example, when calculating solvation or protein–ligand binding free energies, the small-molecule rotamers are expected to influence the result more than any other degrees of freedom. Therefore, the molecular torsions together with center of mass (COM) translation and rotation constitute arguably the most important degrees of freedom for most small molecules. These are also the degrees of freedom that have multiple minima, often separated by high-energy barriers.

**Figure 2:**
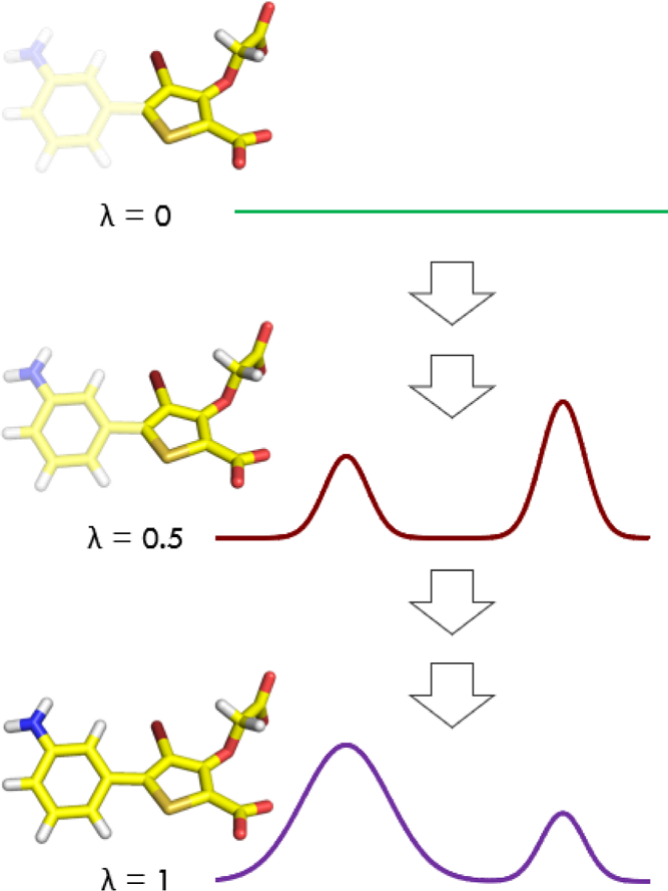
Exploring conformational degrees of freedom with SMC using an alchemical parameter λ. At λ = 0, all of the nonbonded interactions involving the 3-aminophenyl group are fully decoupled, and the distribution of the torsional angle is uniform. At λ = 0.5, the 3-aminophenyl group is partially coupled, and at λ = 1, it is fully interacting, in both cases resulting in two main modes/states.

In these cases, one can use an alchemical approach with a coupling parameter λ, where λ = 0 denotes all relevant interactions turned off and λ = 1 represents the target potential energy function of the system (Figure 2). In this regime, one can readily use any knowledge from the AFE literature. Most notably, an often employed method for deriving the functional form of the intermediate distributions is to introduce a soft-core potential,^47^ which disposes of certain singularities in the potential energy function, thereby improving the statistical efficiency of any estimators dependent on the intermediate λ states. This will be invaluable for the systems discussed later, allowing us to make high-energy insertions and rotations without much of a performance penalty.

There are two common ways to turn on the potential energy interactions: the first is to use the soft-core potential only on the Lennard-Jones (LJ) part of the perturbation, followed by a linear coupling of the electrostatics (“split protocol”), and the second method involves concurrent introduction of all relevant potential terms (“unified protocol”), meaning that a soft-core functional form needs to be used for both LJ and electrostatic interactions. It is expected that a unified protocol is generally less desirable due to the presence of soft-core electrostatic terms, meaning that overlapping positive and negative charges are highly energetically favorable and such unphysical structures can dominate the sampling. On the other hand, the split protocol is expected to produce structures biased toward steric favorability since most of the resampling is expected to take place before introducing the electrostatics. In this work, we will explore and evaluate both protocols.

### 3.2 Adaptively Determining λ_*i*+1_

One can use the knowledge obtained from the distribution of the transition probability weights to assess the quality of the configurational space overlap between the current distribution π(λ_*i*_,*x⃗*) and the next distribution in the sequence π(λ_*i*+1_,*x⃗*). In general, one can use any measure of the distribution overlap to achieve this. In the SMC literature, an overwhelmingly popular metric is the effective sample size (ESS) estimator *R*_ESS_(48)
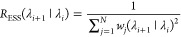
4

A general problem with most overlap metrics is the difficulty in defining what value range can be considered “good”. Although ESS-based measures can be interpreted intuitively more readily than other measures, it has been suggested^49^ that *R*_ESS_ is not necessarily a reliable estimator for the true ESS and should only be seen as a rough heuristic. Instead, one can use a much more conservative measure *R*_min_, which acts as a lower bound for the true ESS^49^
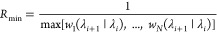
5

After defining the desired system-independent value of this measure, one can iteratively^34,44,46^ determine the next value in the sequence (λ_*i*+1_), which results in an overlap metric closest to this threshold using a basic root finding algorithm, such as bisection. Although each iteration of this adaptive algorithm requires energy evaluations of each walker, they are in practice much faster to perform than generating new samples using dynamics, and the speed of this step will likely be limited by the computational implementation.

The utility of adaptively determining the λ protocol in this way is the guaranteed constant overlap between sequential distributions and the independence of the resulting protocol on the nature of the distributions. Furthermore, if one uses the same overlap metric and value, more dissimilar initial and final distributions will automatically result in a higher number of intermediate distributions without any additional system-specific input.

### 3.3 Adaptively Determining Optimal Sampling Time

An overwhelmingly common way to generate new configurations in biomolecular simulations is MD. This method will be very useful for generating locally decorrelated samples at each λ value. While such decorrelation is not a requirement for the sampling validity of the algorithm, it is in practice desirable to do so since it improves the sample diversity decreased by the resampling procedure. However, the decorrelation time is typically dependent on the system and the nature of the alchemical perturbation. Although it is common practice to choose a value between 1 and 10 ps to achieve local decorrelation, making this step adaptive as well could help maintain the balance between obtaining valid locally decorrelated samples independent of the system and spending as little computational effort as possible.

Since in our typical systems of interest, the equilibrium probability of observing a particular configuration *x⃗*_*j*_ at some λ is solely a function of u(λ,*x⃗*_*j*_), a natural way to measure sample decorrelation is to measure the Pearson correlation coefficient *r*_τ_ between the potential energies of all initial walkers (*u*(λ,*x⃗*_*j*,0_); *j* = 1, ..., *N*) and the walkers decorrelated for τ timesteps (*u*(λ,*x⃗*_*j*,τ_); *j* = 1, ..., *N*). Afterward, the sampling step can only be terminated if *r*_τ_ is within some acceptable range,^46^ for example, |*r*_τ_| ≤ 0.1. In practice, this step also requires an energy evaluation for every walker, and a conceivable implementation could, for instance, involve evaluating these energies every 1 ps, so as to minimize the computational overhead.

### 3.4 Sampling at λ = 0

SMC only converges to the correct distribution at λ = 1 if the initial distribution at λ = 0 has been sampled exhaustively. In a protein–ligand system, this means running long-timescale protein dynamics—a task that itself often requires other sophisticated enhanced sampling methods to produce satisfactory results. An additional problem is the fact that a very small fraction of the generated structures at λ = 0 will typically be relevant at λ = 1 due to the diminishing phase space overlap. In this work, we will not be concerned with long-timescale dynamics, and we will instead explore ligand conformers from a limited set of locally decorrelated equilibrated starting structures. The aim behind this approximation is being able to quickly estimate equilibrium populations biased to the initial structure either as a qualitative tool or as a way to provide information to more expensive methods, such as AFE calculations. Moving beyond this approximation requires a more sophisticated SMC algorithm, which can achieve adequate sampling over time and is thus beyond the scope of this work.

Since this initial stage of SIR is the only checkpoint that generates sample diversity, it is important to take advantage of this. In the test cases we are going to consider, there are three types of sampling moves, for which we know the underlying distribution: torsional rotation, COM rotation, and translation. In all of these cases, we can generate samples typically 1–2 orders of magnitude more than our desired number of walkers due to the fact that all translational and rotational distributions of the noninteracting atoms in these cases are uniform and therefore trivial to sample.

#### 3.4.1 Torsional Rotation

If one removes all nonbonded interactions from at least one side of the torsional bond along with all dihedral terms centered around it, then the initial distribution with respect to the dihedral angle ϕ is uniform, and one can generate configurations by simply drawing random numbers between 0 and 2π. One can use any valid sampling method to achieve this, and in this work, we opt for a low-discrepancy alternative to pseudo-random number generation, which consists of generating equally spaced samples between 0 and 2π with a pseudo-randomly generated offset. In this way, we can be more certain in the representativeness and quality of our samples.

#### 3.4.2 COM Rotation

COM rotation requires all nonbonded interactions between the molecule and the environment to be turned off, and it needs three degrees of freedom to be defined: two spherical coordinate angles on the unit sphere, defining the axis of rotation (θ and ϕ), and the amount of rotation ψ around that axis. To generate uniform rotations on the unit sphere, both ϕ and ψ need to be uniformly distributed between 0 and 2π, while θ = arccos(2*X* – 1) for a uniformly distributed variable X ∈ [0, 1). As in the previous example, one can use different sampling methods to generate the uniformly distributed variables, and although one can couple the different degrees of freedom to reduce the multidimensional sample discrepancy (i.e., sample “clumping”), in this study, we opt for shuffled one-dimensional grid-based samples with a pseudo-random offset for each degree of freedom. Further research will be needed to test alternative low-discrepancy sampling methods for COM rotation.

#### 3.4.3 COM Translation

Much like COM rotation, COM translation requires the molecule of interest to be decoupled from its environment. The simplest case is COM translation within a cuboidal region, in which case only three uniformly distributed random numbers between −1 and 1 are needed to define the new reduced coordinates, which can be then scaled to the dimensions of the region of interest. Alternatively, one can uniformly generate points within a sphere with radius *R*. To achieve this, we can generate the spherical angles θ and ϕ in the same way as in the previous section, while the radius can be expressed as  for a uniformly distributed variable X ∈ [0, 1). Final scaling by *R* results in uniform spherical sampling. Similar considerations about low-discrepancy sampling apply here, and we again opt for the same routine for uniform sample generation as in the previous section.

#### 3.4.4 Coupled Moves

Since in all of our examples, we generate random samples for each degree of freedom independently of the others, this procedure is readily extendable to multiple degrees of freedom. However, the presence of more than a few degrees of freedom can quickly lead to a combinatorial explosion, thereby reducing the sampling efficiency, and in this case, one should consider multidimensional low-discrepancy sampling alternatives. However, this approach is beyond the scope of this work, and we will not be utilizing it.

### 3.5 Using a Conservative Resampling Method

One drawback of SIR is that any loss of walker diversity is irreversible, and in many cases, all of the final samples can be traced to just a few initial samples.^50^ It is important, therefore, to minimize unnecessary diversity loss during the resampling step.

The most obvious way to perform weighted resampling is multinomial resampling with replacement. In this case, one draws each new walker independently from the others. This is problematic since there is always a finite, albeit small, probability that the same sample will be resampled in all cases, resulting in sampling that is potentially not representative of the true weights.

More conservative resampling methods have been proposed, the most deterministic and widely used of which is systematic resampling.^51^ In this case, it is guaranteed that the number of new samples corresponding to each weight *w*_*j*_(λ_*i*+1_|λ_*i*_) (derived from eq 2) is between the rounded-down fractional number and the rounded-up fractional number of walkers *Mw*_*j*_(λ_*i*+1_|λ_*i*_), where *M* is the number of walkers in the next iteration. For example, if the normalized weight of a particular walker is determined to be 0.27 and the total number of walkers in the next iteration is 10, then the fractional number of copies allotted to this walker is 2.7, meaning that systematic resampling will have a 70% probability of copying this walker three times and 30% probability of copying it twice. Because of this certainty, systematic resampling is highly reliable and will be the algorithm of choice in this study.

### 3.6 SMC Workflow in Practice

The first step in describing the problem of interest is identifying the relevant degrees of freedom to be explored, which in turn define a set of interactions to be decoupled at λ = 0. One then supplies an initial structure, the desired number of walkers, and target values for the correlation and decorrelation metrics to the procedure, resulting in an ensemble of structures generated at λ = 1 (Algorithm 1). While the choice of these hyperparameters is somewhat arbitrary and dependent on the available computational resources, they can be used on a variety of systems, and this is the approach that will be taken in this work.
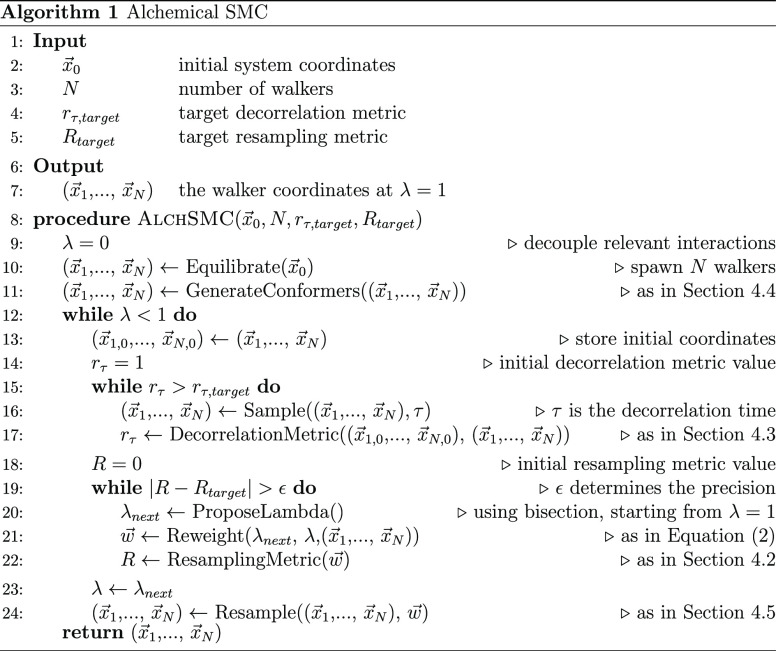


## 4 Methods

### 4.1 System Setup and Simulation

All of the following SMC simulations have been run using OpenMM 7.4.2,^52^ OpenMMTools^53^ 0.19.0, and OpenMMSLICER 1.0.0, a plugin for OpenMM developed during the course of this study, available at https://github.com/openmmslicer/openmmslicer. All proteins were protonated using PDB2PQR^54^ and subsequently parameterized using the ff14SB^55^ protein force field. GAFF2^56^ with AM1-BCC charges^57,58^ was used for all small molecules. All systems were solvated in cubic boxes of TIP3P^59^ water with a length of 3 nm for the solvated ligand systems or 7 nm for the protein–ligand systems. Each system was run independently in six replicates from the same initial coordinates. Each run consisted of an initial minimization, followed by 100 ps of equilibration at λ = 0 before the SMC run. During this equilibration, all protein backbone atoms were harmonically restrained with force constants of 5 kcal mol^–1^ Å^–2^. 500 walkers were used for each replicate with 100 initial conformers generated per walker, where all rotatable bonds between alchemical atoms were rotated in addition to the main alchemical moves. An energy decorrelation condition of |*r*_τ,target_| ≤ 0.1 alongside a minimum relative configurational space overlap of  was consistently used throughout the simulations. These values were arbitrarily chosen with the goal of providing a reasonable balance between the computational cost and sampling quality. Systematic resampling was performed in all cases, and all velocities were resampled from the Maxwell–Boltzmann distribution after each iteration.

All short-range nonbonded interactions had a cutoff of 1.2 nm, while long-range electrostatics were calculated using the particle mesh Ewald method.^60^ A BAOAB^61^ Langevin integrator at 298 K with a 2 fs timestep and a collision rate of 1 ps^–1^ was used, where all water molecules were constrained using the SETTLE^62^ algorithm and all other bonds containing hydrogen atoms were constrained using the SHAKE^63^ and CCMA^64^ algorithms. A Monte Carlo barostat was used for pressure control at 1 atm with rescaling attempts every 50 fs. LJ and electrostatic interactions were switched on either simultaneously (unified protocol) or consecutively from λ = 0 to λ = 0.8 and from λ = 0.8 to λ = 1, respectively (split protocol). A soft-core potential was used for the LJ interactions in both cases and for the electrostatics during the unified protocol with α = 0.5 using the following functional form
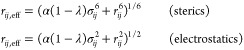
6where all interatom distances *r*_*ij*_ in the potential energy terms involving alchemically modified atoms are replaced with *r*_*ij*,eff_ in the potential energy function and σ_*ij*_ is the “particle size” parameter defined by the LJ potential for the *ij*-th particle pair. In all cases, nonbonded interactions were completely annihilated rather than decoupled from the environment at λ = 0.

SMC was then validated against established methods in one of two ways. The first approach involved a H-REMD simulation in the λ space between 0 and 1 with multiple intermediates defined similarly to SMC. The resulting conformational populations were afterward obtained from the averaged samples at λ = 1. The second approach involved AFE calculations, which were only performed when there were only two expected rotamers separated by a high kinetic barrier. In this setting, two separate perturbations were performed in a single-topology fashion from both initial conformations, where the only difference was the rotation of the relevant torsion by 180°, to the nearest common physical intermediate (i.e., to propene in the case of butene and to a phenyl group in place of a substituted phenyl group). The corresponding dihedral terms were not scaled during the AFE calculations so that no unwanted transitions between the rotamers of interest would be observed. The population ratio between both rotamers  was then calculated using the formula *kT* ln  = Δ*G*_state1→intermediate_^⊖^ − Δ*G*_state2→intermediate_^⊖^.

Both AFE and H-REMD calculations were performed in sextuplicate in GROMACS^65^ 2018.4 patched with PLUMED^66^ 2.4.3 using ProtoCaller^67^ 1.1 from the same initial structures as those used for the SMC runs (and in the case of AFE, the relevant manually generated rotameric states). In all cases, the alchemically decoupled groups in the H-REMD simulations were the same as those in the SMC simulations. The only exceptions were the T4-lysozyme/3,5-difluoroaniline simulations, where a single ligand carbon atom remained coupled at λ = 0 to prevent diffusion away from the (closed) binding site without the need of additional restraint potentials. In some cases, several batches of H-REMD simulations were run from different starting conformations to investigate initial structural biasing. These will be indicated later in the text.

The split alchemical protocol was used during both AFE and H-REMD calculations, with 30 initial λ windows used for co-perturbing the soft-core sterics and the bonded interactions and 10 subsequent windows for the electrostatics. All λ values were equally spaced to two significant figures, except for the initial values, which were more closely spaced in an attempt to increase the phase space overlap: 0.001, 0.01, 0.02, 0.03, and 0.05. The Bennett acceptance ratio^68^ was used for free energy analysis with snapshots every 5 ps.

The AFE protocol involved an initial 25,000-step steepest descent minimization, followed by a 50 ps *NVT* equilibration and a 50 ps *NPT* equilibration before a 4 ns *NPT* production. The Berendsen barostat^69^ was used for equilibration in all cases, while the Parrinello–Rahman barostat was used for the production runs.^70^ The LINCS algorithm^71^ was used to constrain the non-water hydrogen atoms during both stages, while the rest of the simulation settings matched the ones from the SMC runs. In the H-REMD simulations, the above equilibration schedule was only performed at λ = 1, and the resulting volume was fixed for all replicas. This was followed by an additional minimization and equilibration only in the *NVT* ensemble and subsequent 4 ns simulations at constant volume. During both H-REMD equilibration and production, adjacent replica swaps were attempted every 1 ps.

### 4.2 Analysis

All of the measured populations in this study were weighted by the estimated partition function ratio  for the relevant simulation, as previously described in eq 3. These were used to report weighted averages and weighted sample standard deviations. Since the latter can be low even when there is a high spread of data due to large discrepancies in the replicate weights, all replicate data points will also be added to the plots to visualize the unweighted spread of the resulting values between the runs. On the other hand, the estimated dimensionless free energies and the simulation times have been reported as unweighted averages with unweighted standard deviations in the main text.

To appropriately analyze the relevant kinetically separated states, clustering on the degrees of freedom of interest was performed. In most cases, this was achieved using manually defined cluster boundaries determined from the observed multimodal distributions of the angle of interest. The only exception is the ligand common core clustering analysis performed for transforming growth factor β (TGF-β), where all trajectories at λ = 1 from the SMC and H-REMD simulations were pooled together and aligned against the protein backbone α-carbon atoms of the initial structure using MDTraj^72^ and MDAnalysis.^73,74^ Afterward, the three Euler angles providing the best alignment of the common core ligand atoms against their initial coordinates were calculated using the align_vectors routine implemented in SciPy.^75^ The sines and cosines of these three Euler angles (six degrees of freedom in total) were used to perform agglomerative clustering with default settings, as implemented in scikit-learn.^76^ This analysis resulted in two clusters, whose populations will be reported later in the text alongside two representative structures corresponding to each cluster.

Where applicable, the number of round trips of the H-REMD simulations has been reported. These have been calculated as the total number of round trips of all replicas, where a round trip denotes the transition from λ = 1 to λ = 0 and back of a single replica.

In the following results, sampling times have been reported as the aggregate time of all λ windows (AFE and H-REMD) or the total of all walkers (SMC).

## 5 Results

### 5.1 Butene in Water

**Figure 3:**
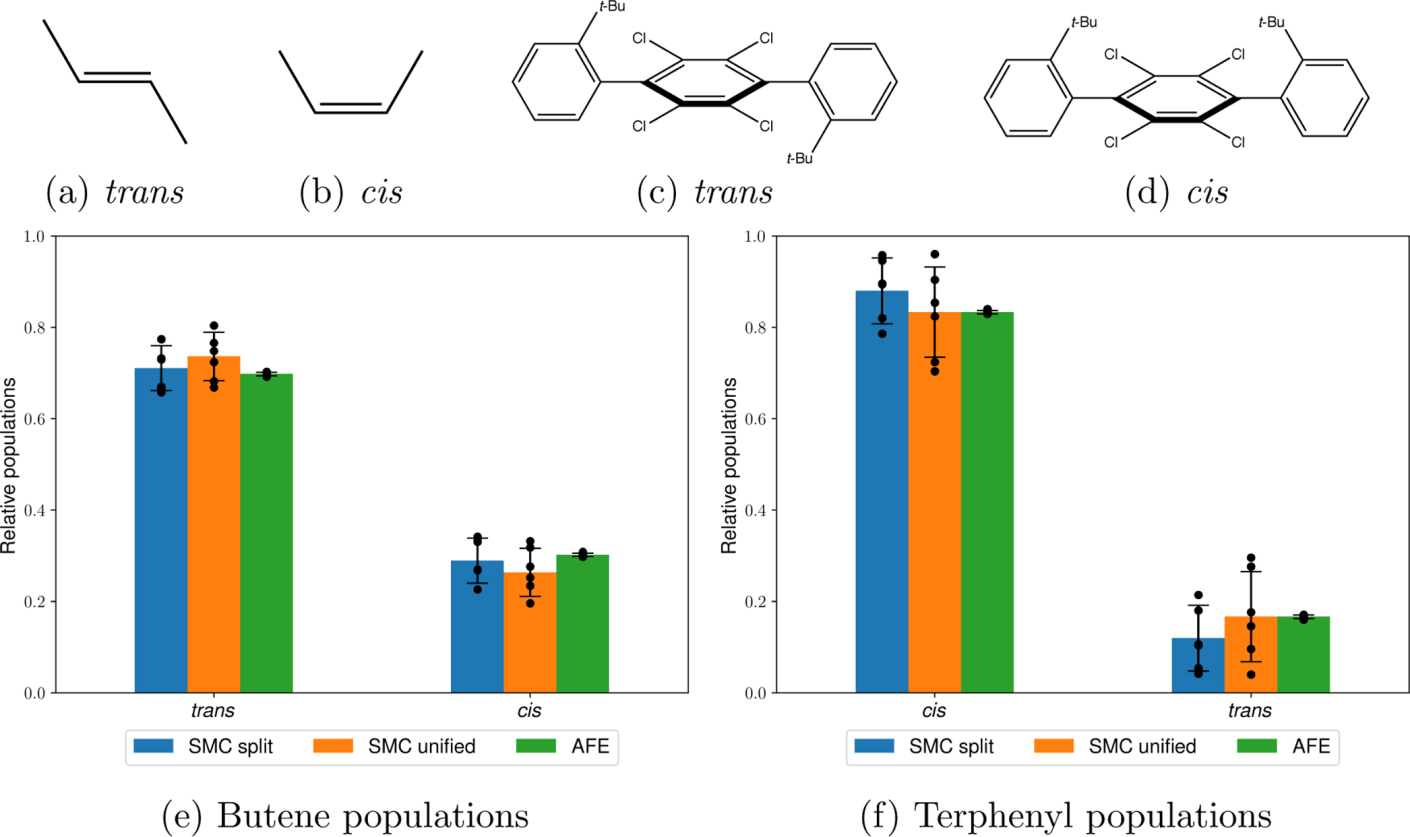
Two butene stereoisomers (a,b) and the two isomers of the terphenyl derivative (c,d) with populations measured via AFE and SMC (e,f) using the split and unified protocols. The heights of the bars represent the mean values weighted using the estimated partition function ratio , and the error bars represent one weighted standard deviation based on six independent runs (shown as individual data points), as described in Section 4.2.

One of the simplest systems involving a high kinetic barrier is the cis–trans isomerization of butene solvated in water (Figure 3). Although not of significant practical interest, this test case is a good demonstration of SMC’s capabilities in an ideal setting. To explore this kinetic barrier, all atoms on one side of the double bond, together with all corresponding dihedral terms, were decoupled from their environment at λ = 0. This enabled us to directly sample this dihedral angle from the uniform distribution at λ = 0.

The results from SMC using both the unified and split protocols are presented in Figure 3e. Both protocols compare favorably to the converged 160 ns AFE results (70 ± 0% trans), with the split protocol resulting in 71 ± 5% and the unified protocol yielding an average of 74 ± 5%. In addition, both protocols result in similar performances, with 16 ± 1 ns total computational for the adaptive split protocol and 15 ± 1 ns for the adaptive unified protocol. Finally, both protocols result in comparable standard deviations of −ln *Z*_1_/*Z*_0_ (here and henceforth referred to as “dimensionless free energy”) with values of 4.79 ± 0.28 and 4.89 ± 0.15 for the split and the unified protocols, respectively, indicating good convergence in both cases.

### 5.2 Terphenyl in Water

A much more challenging test case with an insurmountable kinetic barrier is the terphenyl derivative shown in Figure 3. It is expected that only alchemical methods can handle such a system since approaching the kinetic barrier with all interactions turned on will result in large repulsive forces and numerical instability. Moreover, alchemically decoupling the *tert*-butylphenyl substituent is also likely to be challenging, making this system a good example of a difficult enhanced sampling problem in solution. Similar to the previous test case, one of the tert-butylphenyl substituents, as well as all dihedral terms corresponding to the rotatable bond, was completely decoupled at λ = 0 to facilitate sampling.

Figure 3f demonstrates that both the split and unified protocols yield similar results for the main cis conformer: 87 ± 7 and 83 ± 9%, respectively, compared to 83 ± 0% using 160 ns AFE. Moreover, both methods estimate the dimensionless free energy very precisely: 35.60 ± 0.23 for the split protocol and 35.64 ± 0.19 for the unified protocol, indicating good sampling consistency between the SMC alchemical protocols and repeats. Finally, both methods show similar performance, with the split protocol being slightly slower on average (42 ± 2 ns) than the unified protocol (37 ± 3 ns). The longer average simulation times compared to that of the butene perturbation show that the adaptive protocol with the same hyperparameters automatically allocates more computational time to a more difficult problem, as expected.

### 5.3 T4-Lysozyme/*p*-Xylene

**Figure 4:**
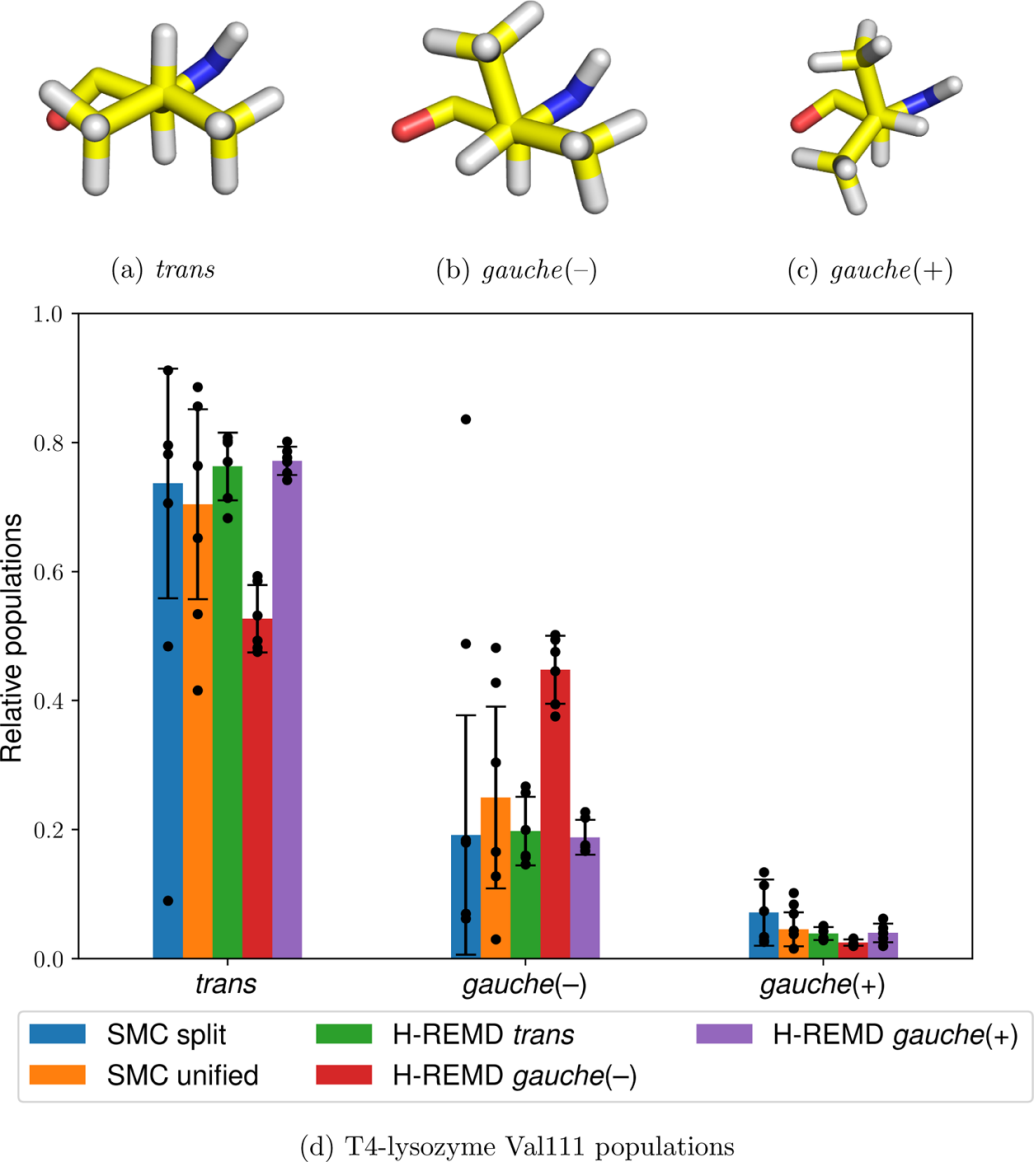
Three Val111 rotamers (a–c) in T4-lysozyme/*p*-xylene and the relative populations of all states using split and unified SMC and H-REMD from the three different initial rotamers (d). The heights of the bars represent the mean values weighted by the estimated partition function ratio , and the error bars represent one weighted standard deviation based on six independent runs (shown as individual data points), as described in Section 4.2.

A seemingly simple case that nevertheless showcases the inability of regular MD to provide adequate sampling is the exploration of the active site Val111 rotamers (Figure 4a–c) in model T4-lysozyme L99A with bound *p*-xylene (PDB ID: 187L(77)). It has previously been shown^78^ that MD results in highly insufficient rotamer transitions even at 1 μs, suggesting that enhanced sampling is indispensable for this system. We can handle this system similarly to the previous test cases by completely decoupling the Val111 isopropyl group and the corresponding dihedral term to facilitate movement at λ = 0. In this setting, the sampling of *p*-xylene was not enhanced.

The resulting SMC protocols are highly efficient, requiring an average of 25 ± 2 and 21 ± 2 ns per repeat for the split and unified protocols, respectively, while exploring all relevant Val111 rotamers. Although the split protocol results in higher variance than the unified protocol (Figure 4d), both methods result in similar torsional populations and are qualitatively consistent with one another. This is also demonstrated by the relatively high precision of the dimensionless free energy: −43.09 ± 0.85 and −44.32 ± 0.72 for the split and unified protocols, respectively.

To test the accuracy of the results, they were compared against six H-REMD simulations from each initial Val111 conformer (18 simulations in total) with 160 ns per repeat, or 4 ns per replica. As shown in Figure 4, even after an average of 252 ± 13 round trips per repeat, there is a significant bias in the populations depending on the starting conformation. This discrepancy can be partially attributed to the fact that the H-REMD implementation used does not explicitly draw the decoupled dihedral from the uniform distribution at λ = 0 but instead relies purely on integrator decorrelation to achieve this, meaning that any Val111 state transitions are effectively slowed down even when there are no kinetic barriers. In contrast, the SMC simulations are not biased toward the initial Val111 conformation since all simulations start from a completely decoupled state. Nevertheless, the relative ranking of the populations is consistent between different starting structures, as well as with the SMC simulations using either the split protocol or the unified protocol. Although the predicted dominant rotamer (trans) does not correspond to that in the crystal structure [gauche(−)], the agreement between both enhanced sampling methods suggests that this discrepancy is most likely related to the force field quality and/or long-timescale populations shifts due to, for example, protein rare events, which are beyond the scope of this work.

### 5.4 T4-Lysozyme/3,5-Difluoroaniline

A more difficult test case is coupling the Val111 motion with translational and rotational movements of the ligand. An example ligand is 3,5-difluoroaniline bound to a L99A/M102Q T4-lysozyme mutant. In this case, the ligand was completely decoupled in addition to the Val111 isopropyl group and uniformly moved at λ = 0 within a sphere with a radius of 0.5 nm centered on its initial COM, suggested by the crystal structure (PDB ID: 1LGX(79)). Since there were two competing ligand binding modes in the electron density, the one with the higher experimentally determined occupancy was chosen for the initial COM evaluation.

**Figure 5:**
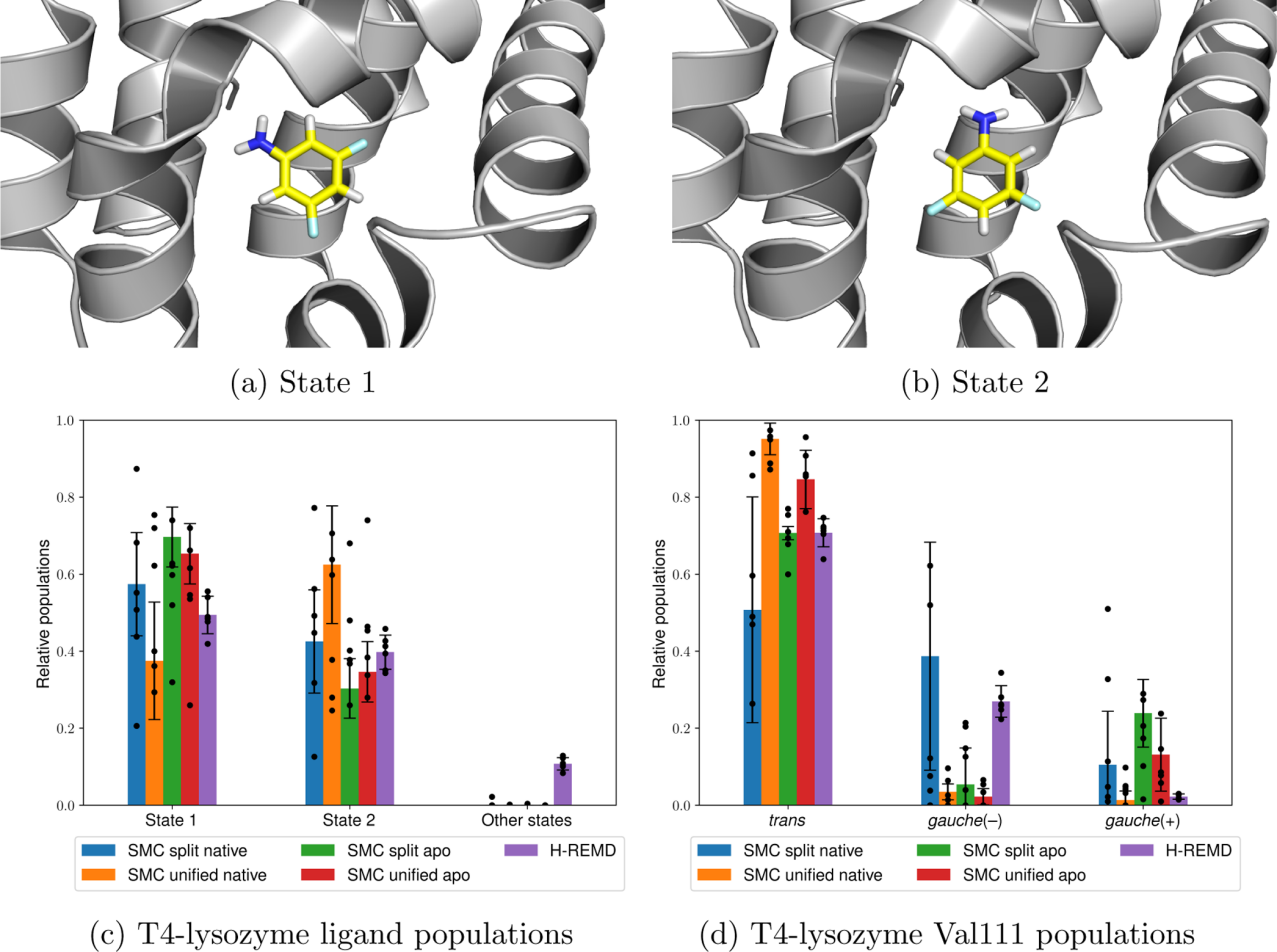
Two 3,5-difluoroaniline binding modes (a,b) bound to T4-lysozyme, the relative populations of both ligand states using the split and unified SMC protocols and H-REMD (c), and the Val111 states from the same simulations (d). The heights of the bars represent the mean values weighted by the estimated partition function ratio , and the error bars represent one weighted standard deviation based on six independent runs (shown as individual data points), as described in Section 4.2.

The SMC simulations required an average of 48 ± 2 ns simulation time for the split protocol and 40 ± 2 ns for the unified protocol. Both protocols resulted in two main binding modes for the ligand, which are shown in Figure 5. The states are approximately equally probable, with acceptable agreement between the split protocol (57 ± 13: 43 ± 13%), the unified protocol (38 ± 15: 62 ± 15%), 160 ns H-REMD (49 ± 5: 40 ± 4%), and experiment (60:40%). The dimensionless free energies are also consistent, averaging −266.32 ± 3.46 for the split protocol and −266.68 ± 2.27 for the unified protocol.

**Figure 6:**
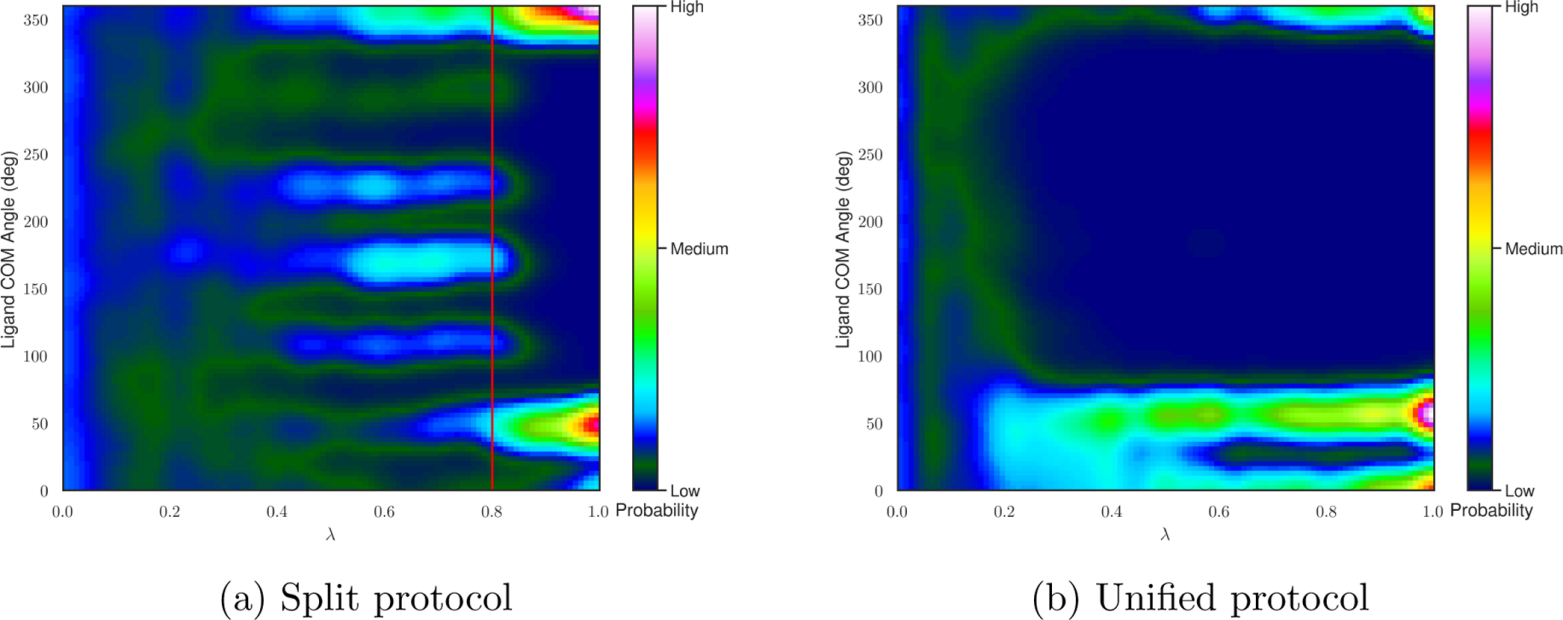
Heat maps of 3,5-difluoroaniline COM angle populations relative to the initial dominant conformer using the split (a) and unified (b) SMC protocols taken from a single representative repeat. The data at discrete λ values have been smoothed in both cases for visual purposes. The solid red line in (a) indicates the alchemical intermediate with fully coupled sterics and fully decoupled electrostatics.

It is interesting to note the sampling differences between both SMC protocols during the intermediate λ values. As shown in Figure 6a, the split protocol explores six different binding modes with approximately equal probabilities during the steric coupling step before collapsing into the two main binding modes during the electrostatic coupling step. In contrast, the unified protocol (Figure 6b) collapses almost immediately into the two main binding modes, indicating that in this case, there is higher monotonicity in the population changes over λ.

The same SMC protocols were performed on the same mutant using a different crystal structure (PDB ID: 1LGU(79)), where only mercaptoethanol (part of the crystallization liquor) was bound, making this crystal structure the closest experimentally available structure to an apo form for this mutant. Little difference in the results was observed using both the split protocol (70 ± 8: 30 ± 8%) and the unified protocol (65 ± 8: 35 ± 8%), indicating that the method is not strongly dependent on the initial crystal structure in this case and the results are therefore not biased in an obvious way.

Larger differences were observed for the Val111 rotamers, where there were discrepancies between the populations from both SMC protocols and H-REMD. Since both the native and the apo structures exhibit significant differences between both protocols, it can be concluded that the split and unified protocol results are not consistent with each other in this case. This can be attributed to the different ways in which the different λ schedules affect the time-dependent dynamics of each walker. Since the simulation time for each walker remains very short, the lack of long-timescale sampling can therefore result in biased populations.

### 5.5 Protein Tyrosine Phosphatase 1B

**Figure 7:**
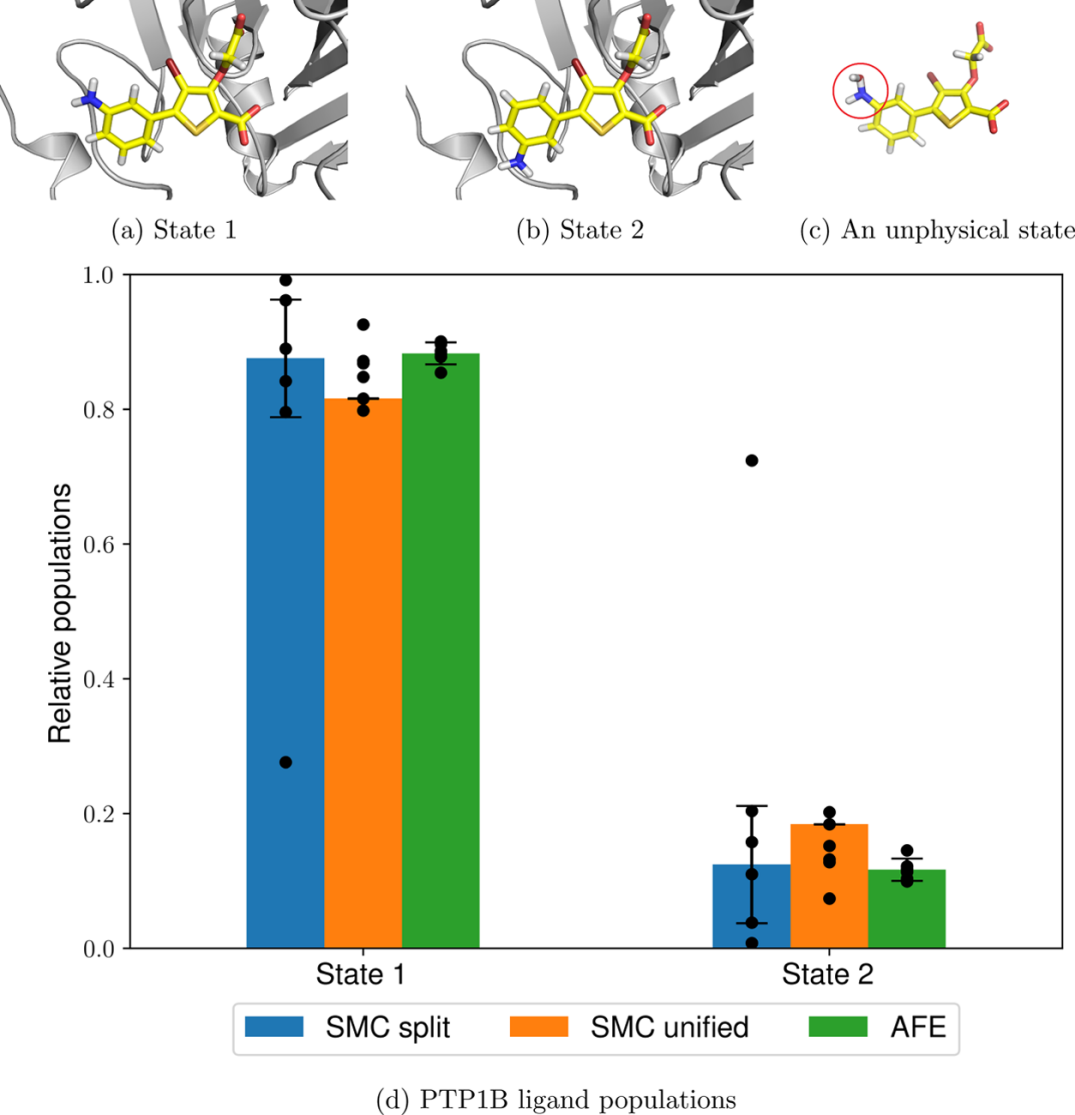
Two thiophene derivative rotamers bound to PTP1B (a,b), unphysical interactions between the amino group and a solvent water molecule commonly observed during the unified protocol (c) (circled in red), and the relative populations of both states using SMC and AFE calculations (d). The heights of the bars represent the mean values weighted by the estimated partition function ratio , and the error bars represent one weighted standard deviation based on six independent runs (shown as individual data points), as described in Section 4.2.

Another commonly encountered problem is handling dihedral rotations of flexible bound ligands, such as the thiophene derivative bound to protein tyrosine phosphatase 1B (PTP1B) (PDB ID: 2QBS(80)), as shown in Figure 7. In this case, there are two main states of interest (Figure 7a,b), and we can explore this rotation by completely decoupling the 3-aminophenyl group and the relevant dihedral terms at λ = 0.

Similar to the previous torsional rotation cases, there is a good agreement between the dominant conformer in the split protocol (88 ± 9%), the unified protocol (81 ± 0%), and AFE (88 ± 2%) and the experimental crystal structure. However, in this case, the split protocol results in a much higher unweighted standard deviation (26%), mostly caused by a single outlier. Although the split protocol performs apparently worse than the unified protocol, the latter exhibits extremely poor and variable dimensionless free energy differences: 231.73 ± 20.56 compared to −85.51 ± 2.30 for the former. Since the dimensionless free energies correspond to the negative logarithm of the average relative weight of all walkers sampled from a particular simulation, the unified protocol has a negligible total weight compared to the split protocol due to its strikingly high dimensionless free energy. Therefore, even though the dihedral profiles yielded by the unified protocol appear consistent, the sampling is nevertheless remarkably poor. This can be explained by an energetically favorable overlap between one of the ligand nitrogen atoms and a water hydrogen atom, coupled with an interaction between the aniline hydrogen and the water oxygen (Figure 7c). These unphysical interactions are not forbidden and quite favorable since introducing a soft-core potential to both sterics and electrostatics removes all potential energy singularities at the atom centers. Although these interactions vanish at λ = 1, they persist for most of the λ schedule, meaning that in this case, the split protocol is much more preferable. This conclusion is also supported by the average simulation times: 42 ± 2 ns for the split protocol and 55 ± 1 ns for the unified protocol, indicating that these unphysical states hinder the short-timescale dynamics as well.

### 5.6 Transforming Growth Factor β

**Figure 8:**
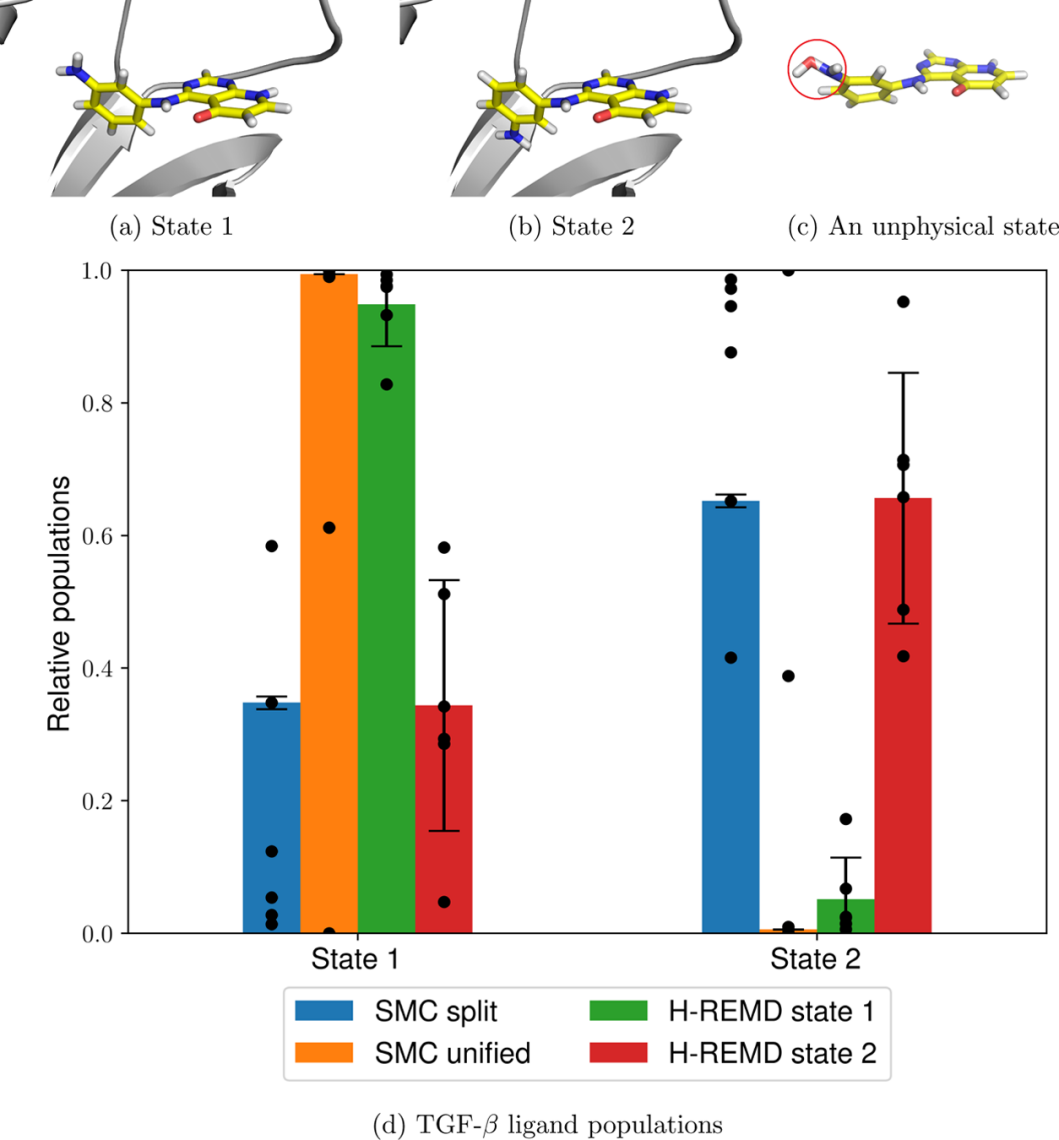
Two TGF-β ligand rotamers (a,b), unphysical interactions between the amino group and a solvent water molecule commonly observed during the unified protocol (c, circled in red), and relative populations of both states using the split and unified protocols and H-REMD starting from either of the states (d). The heights of the bars represent the mean values weighted by the estimated partition function ratio , and the error bars represent one weighted standard deviation based on six independent runs (shown as individual data points), as described in Section 4.2.

**Figure 9:**
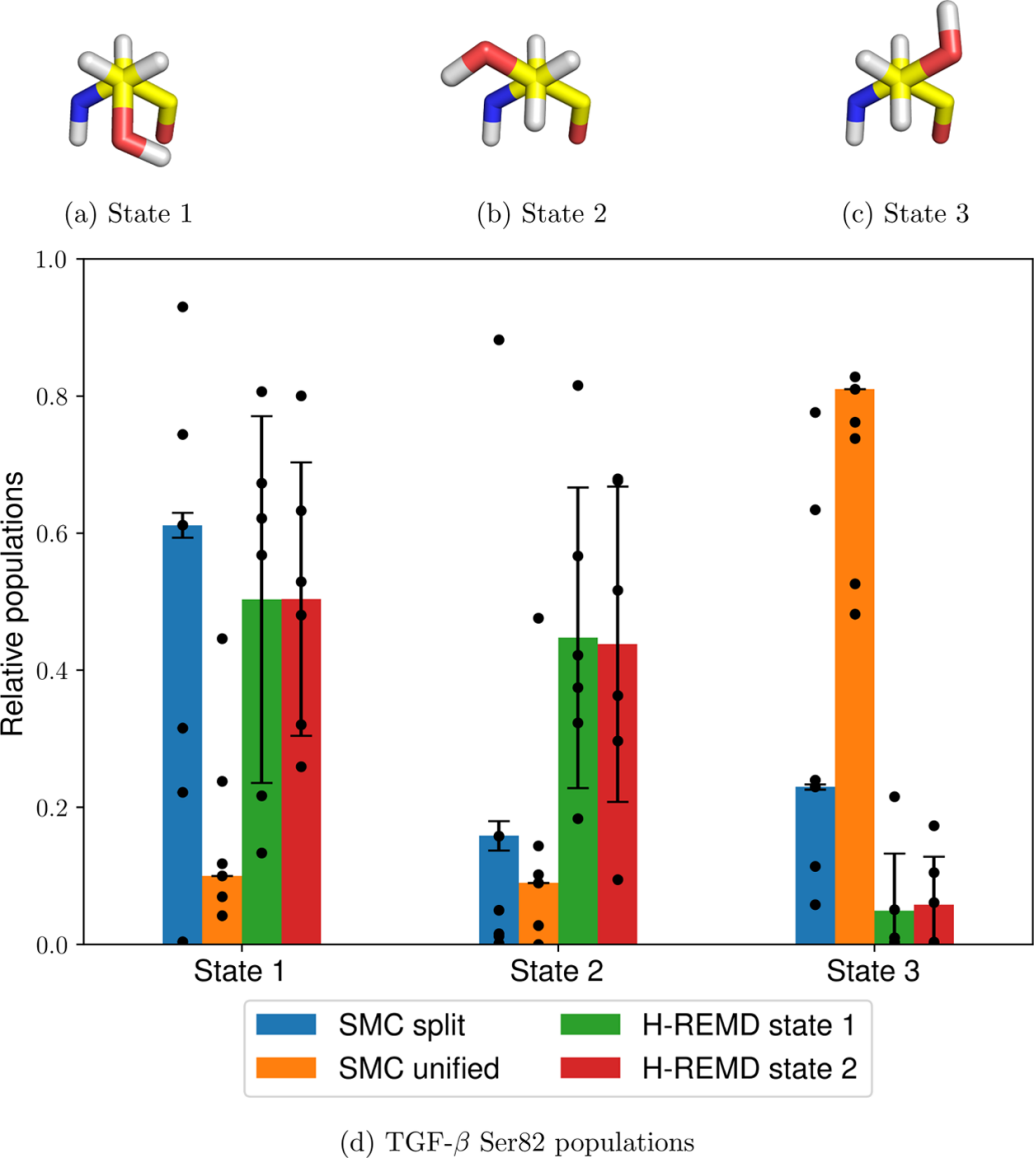
Three TGF-β Ser82 rotamers (a–c) and relative populations of all states using the split and unified protocols and H-REMD starting from either of the states (d). The heights of the bars represent the mean values weighted by the estimated partition function ratio , and the error bars represent one weighted standard deviation based on six independent runs (shown as individual data points), as described in Section 4.2.

The final test case combines a torsional rotation of a flexible ligand bound to TGF-β and a nearby Ser82 side chain rotation. In this case, we have used the initial protein coordinates of TGF-β bound to a ligand containing a related symmetric 4-aminophenyl substituent (PDB ID: 4X2G(81)) combined with the initial binding mode of the 3-aminophenyl-substituted ligand of interest (PDB ID: 4X2J(81)) so that the potential bias toward a particular conformer in the initial PDB file has been minimized. It is known from the PDB file that there are two approximately equally populated alternative conformations of the ligand (Figure 8a,b) and the nearby Ser82 residue (Figure 9a,b). As with the previous examples, this system was handled by decoupling the 3-aminophenyl ligand group concurrently with the Ser82 hydroxymethyl group.

Similarly to PTP1B, the unified protocol has sampling difficulties related to favorable unphysical interactions between an alchemically modified amine group and a water molecule (Figure 8c), resulting in large discrepancies between the dimensionless free energies: −225.51 ± 6.25 for the split protocol, compared to 200.65 ± 49.12 for the unified protocol, showing once again that this type of interaction results in populations with a negligible total weight compared to those obtained from the split protocol. Another point of similarity to the previous test case is the higher average simulation time that is needed by the unified protocol: 90 ± 3 versus 60 ± 6 ns for the split protocol.

In both cases, however, there is a marked increase in the relative weight variance compared to the previous test cases, indicating poor convergence. This is also confirmed by the ligand dihedral profiles (Figure 8d), which show significant quantitative and qualitative differences between the results of both protocols. This observation is reflected by the low efficiency of the 160 ns H-REMD runs, with an average of only 7 ± 4 round trips per repeat. Despite the low number of round trips and the slow convergence, the data from the H-REMD simulations starting from both ligand rotamers suggest that the first conformer (Figure 8a) is likely more stable than the other, implying that the unified protocol is surprisingly qualitatively consistent with H-REMD. The two SMC protocols and H-REMD do not agree on the Ser82 populations, however (Figure 9d), meaning that in both cases, there is evidence for insufficient sampling.

**Figure 10:**
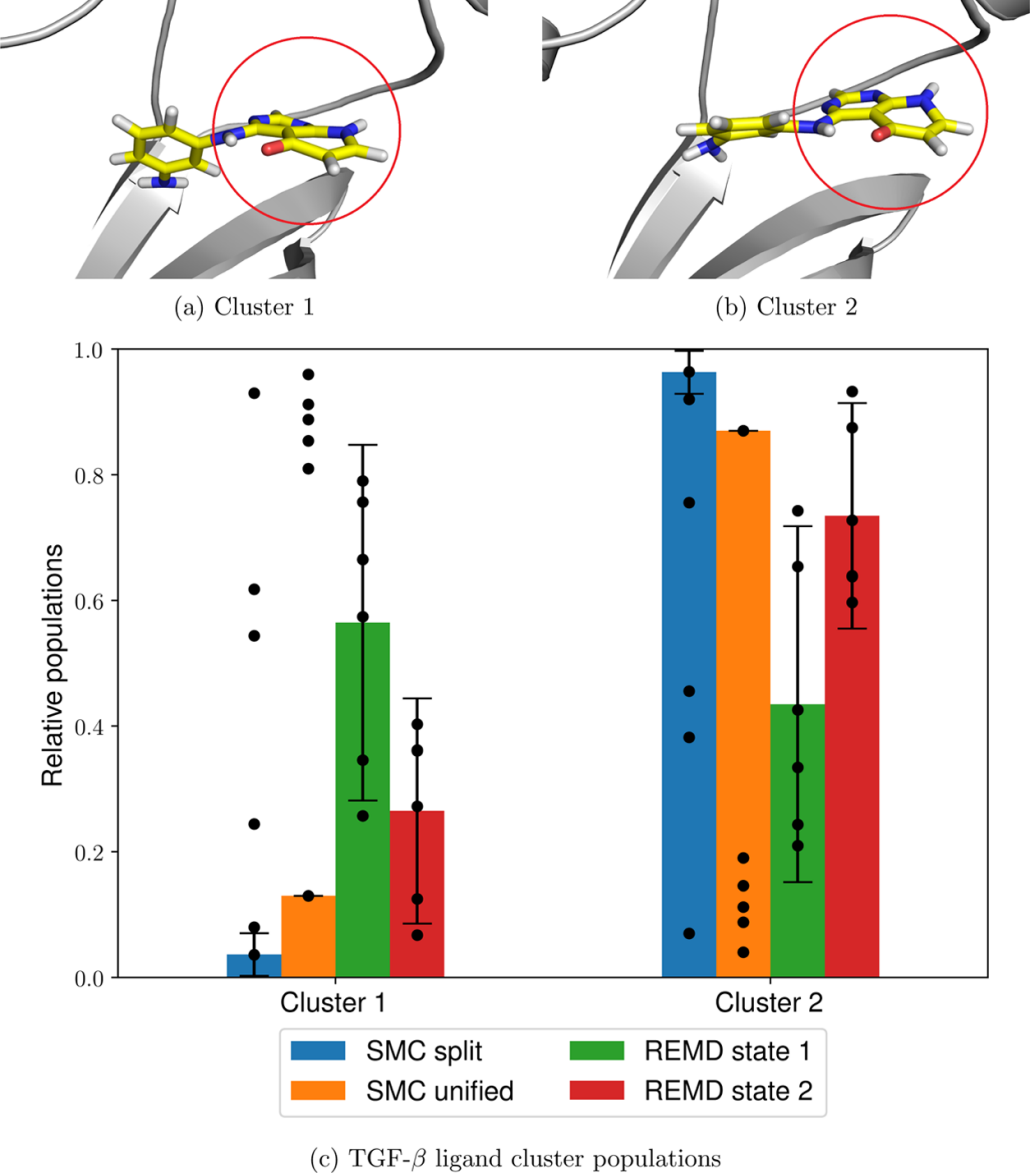
Two TGF-β ligand clusters (a,b, common core circled in red) and their relative populations using the split and unified protocols and H-REMD starting from either of the states (c). The heights of the bars represent the mean values weighted by the estimated partition function ratio , and the error bars represent one weighted standard deviation based on six independent runs (shown as individual data points), as described in Section 4.2.

Clustering analysis of the ligand common core at λ = 1 using agglomerative clustering (as described in Section 4) reveals the presence of two distinct, albeit apparently similar, clusters, which correspond to a concerted translational and rotational motion of the ligand common core (Figure 10a,b). It can be seen that the first cluster is overrepresented in the unified protocol structures, as well as the H-REMD simulations starting from the dominant state (Figure 10c). However, the second cluster is the one resulting in the highest total relative weights for both the split and unified SMC runs. Since both clusters are more equally sampled during the comparatively longer H-REMD simulations, this behavior indicates an insufficient level of decorrelation of the SMC results from the initial structure, resulting in significant biasing of the observed ligand dihedral populations. Moreover, these ligand transitions present an orthogonal rare event that is not adequately sampled even at longer timescales and thus results in increased population variance for both SMC and H-REMD.

## 6 Discussion

The above results show that SMC is extremely efficient at exploring ligand conformers in solution, even for alchemical changes that would be considered difficult to perform in practice. This is not surprising since this is the ideal setting for the method: the ligand degrees of freedom are the only ones that require extensive sampling, while the environment does not need much long-timescale sampling to respond to the ligand motions. Therefore, SMC can be a valuable tool in exploring the degrees of freedom of solvated small molecules and is likely one of the most robust ways to achieve this.

The T4-lysozyme test cases show that a closed binding pocket exhibiting little flexibility also constitutes a favorable application of the method. We have shown that SMC is unaffected by high kinetic barriers and relatively unbiased toward the initial ligand structure, while providing efficient protocols that require no system-specific parameters. These results appear to hold even when exploring coupled motions between a side chain and a ligand.

Similar observations have been made for PTP1B, where the ligand is strongly bound to the protein and the rotatable group of interest faces the solvent. In this case, the efficiency of SMC is similar to that observed in the solvated ligand systems. However, the resulting unweighted population variances from all protein test cases are much higher compared to those from the first two test cases, and this trend carries to the dimensionless free energies. This is expected since protein–ligand systems present a much more challenging sampling problem compared to solvated ligand systems.

TGF-β presents a more challenging system, where rare motions of the unmodified part of the ligand contribute to a much higher observed dihedral population variance than in the previous test systems. This behavior is observed for both SMC and H-REMD, meaning that exploring long-timescale motions for this system is crucial and the short-timescale SMC runs are not sufficient in this case. It is therefore important to be able to identify such potentially problematic systems a priori, and this should be addressed in future work.

The above test cases also show the advantages and disadvantages of the split and unified force field scaling protocols. It has been demonstrated that the unified protocol can result in an unpredictable performance and can suffer from unphysical interactions between atoms with opposite charges, resulting in them collapsing on top of one another. This means that while the unified protocol can in many cases be more efficient than the split protocol, it is also less robust. The split protocol, on the other hand, has been shown to be extremely consistent both in terms of sampling time and free energy estimation but often results in a larger unweighted variance of the sampled populations. It is not yet clear how the above protocols will perform in a system that exhibits a drastic shift in rotamer populations when the electrostatic interactions are switched on, but the above results strongly suggest that the split protocol is by far the safer and more conservative choice for most systems.

All of the above results paint a clear picture of the current advantages and limitations of SMC. SMC excels in cases where one is interested in few degrees of freedom and where the populations of interest remain relatively unchanged over long timescales. In such systems, one can expect high performance with minimal user input, meaning that very different systems can be run with the same hyperparameters without external intervention. Another advantage of SMC is the lack of a need for supplying an initial conformation of the degree of freedom of interest, thereby providing an unbiased estimate of the population over this degree of freedom. In contrast, while H-REMD results in populations with apparently lower variance than SMC, it also exhibits a long-timescale bias toward the initial conformation. Taking this bias into account then results in a similar performance to that of SMC. Moreover, the collective estimated SMC simulation weights  provide a straightforward way to measure sampling quality, while investigating bias is not as obvious, meaning that SMC is more useful for performing exploratory simulations. On the other hand, AFE calculations result in significantly lower variance than both SMC and H-REMD, but their main disadvantage is that a separate simulation is required for each cluster of interest. These need to be known in advance, and this knowledge is not always available in practice.

SMC is therefore a valuable qualitative exploratory tool, which can quickly provide initial structures that are unbiased over a particular degree of freedom, as well as generate an efficient λ schedule for another more computationally expensive method, such as AFE calculations or H-REMD simulations. The latter methods, on the other hand, can sample over arbitrarily long timescales, thereby being systematically improvable while simultaneously reducing their bias toward the initial protein crystal structure over time. This decorrelation is crucial in, for example, binding free energy calculations, where the initial protein crystal structure can significantly impact the calculated free energies.^82^ Therefore, one can use the strengths of both SMC and long-timescale methods to minimize the dependence of the sampled conformations on the choice of initial protein and ligand coordinates.

Owing to the shortness of its simulations, SMC is so far impractical for sampling long-timescale motions and can suffer from initial structure templating, as well as kinetic trapping, which occurs during the alchemical steps. The latter is a significant challenge not only for SMC but also for H-REMD, and it can be triggered by orthogonal rare events, such as slow motions of the unmodified part of the ligand, which makes this behavior difficult to predict. These problems will therefore require key modifications to the SMC method and will be the subject of future work.

## 7 Conclusions

We have presented an alchemical version of SMC, a directed irreversible method that can be used for sampling rare events using adaptive importance resampling. Alchemical SMC combines adaptive SMC methods with the knowledge from the AFE literature and is thus ideally suited for protein–ligand and related systems, systems where the requirement for system-specific method parameters would be highly undesirable.

The performance of alchemical SMC was demonstrated in a variety of test cases where regular MD is unable to provide adequate sampling, and we have also measured the relative efficiencies of a split perturbation protocol and a unified scheme, where steric and electrostatic interactions are coupled separately and concurrently, respectively. Our results show that SMC performs best when the results are largely independent of long-timescale motions and other important orthogonal kinetic barriers. In these cases, SMC provides efficient sampling and is unaffected by the exact nature and size of the system. The most consistent and robust results are also observed when the split protocol is used, and we recommend it over the unified protocol for the general case.

We have shown that alchemical SMC is good at generating unbiased conformations over a selection of degrees of freedom. Moreover, it provides a good metric for convergence, the estimated collective weight , which can be used to assess the sampling quality over different simulation repeats. In this setting, methods such as H-REMD are less useful due to their long-timescale bias, which is often difficult to detect. Similarly, AFE calculations require prior knowledge for the conformers of interest, and their cost scales rapidly with the number of possible states for each degree of freedom. This makes alchemical SMC a good method for performing exploratory simulations with minimal input.

In one of the test cases, SMC exhibits large variance and poor convergence, and this suboptimal performance can be attributed to high dependence on the initial coordinates, meaning that the method needs to be extended to long-timescale sampling. This will be the subject of future research.

## Supporting Information Available

Raw data reported in the publication (XLSX)
Initial structures and Python scripts used to run all simulations using OpenMMSLICER and ProtoCaller (ZIP)
