# Supplementary material for: Enhancing Ligand and Protein Sampling Using Sequential Monte Carlo
Source: J Chem Theory Comput. 2022 May 19;18(6):3894–910. doi: 10.1021/acs.jctc.1c01198 (PMC9202307; doi:10.1021/acs.jctc.1c01198)
Supplement: Supplementary file 2 — ct1c01198_si_002.zip [file ct1c01198_si_002.zip › Authors.pdf]

Enhancing Ligand and Protein Sampling Using Sequential Monte Carlo

Miroslav Suruzhon,[†] Michael S. Bodnarchuk,[‡] Antonella Ciancetta,[¶,§] Ian D. Wall,[‖] and Jonathan W. Essex[*,†]

†*School of Chemistry, University of Southampton, Highfield, Southampton SO17 1BJ, United Kingdom*

‡*Computational Chemistry, R&D Oncology, AstraZeneca, Cambridge CB4 0WG, United Kingdom*

¶*Sygnature Discovery, Bio City, Pennyfoot St, Nottingham NG1 1GR, United Kingdom*

§*Department of Chemical, Pharmaceutical and Agricultural Sciences—DOCPAS, Via Fossato di Mortara 17/19, 44121, Ferrara—University of Ferrara, Italy*

‖*GSK Medicines Research Centre, Gunnels Wood Road, Stevenage SG1 2NY, United Kingdom*

E-mail: J.W.Essex@soton.ac.uk
